# Design and Analysis of the Capacitive RF MEMS Switches with Support Pillars

**DOI:** 10.3390/s22228864

**Published:** 2022-11-16

**Authors:** Hongbo Feng, Jiabin Zhao, Chengsi Zhou, Mingxin Song

**Affiliations:** School of Applied Science and Technology, Hainan University, Haikou 570228, China

**Keywords:** RF MEMS, switches, impact velocity, pull-in time, reliability

## Abstract

Conventional parallel capacitive RF MEMS switches have a large impact during the suction phase. In general, RF MEMS switches have to be switched on and off in a considerably fast manner. Increasing the driving voltage enables fast switching but also increases the impact force, which causes the beam membrane to be prone to failure. In the present study, the addition of two support pillars was proposed for slowing down the fall of the beam membrane based on the conventional RF MEMS parallel switch, so as to reduce the impact velocity. As such, a novel RF MEMS switch was designed. Further, simulation software was used to scan and analyze the positioning and height of the support pillars with respect to electromechanical and electromagnetic performance. The simulation results show that the optimal balance of impact velocity and pull-in time was achieved at a height of 0.8 um, a distance of 10 um from the signal line, and an applied voltage of 50 V. The impact velocity was reduced from 1.8 m/s to 1.1 m/s, decreasing by nearly 40%. The turn off time increased from 3.9 us to 4.2 us, representing an increase of only 0.05%. The insertion loss was less than 0.5 dB at 32 GHz, and the isolation was greater than 50 dB at 40 GHz.

## 1. Introduction

Due to their low power consumption, high isolation, low insertion loss, and ability to integrate with other electronic devices, RF MEMS (Radio Frequency Micro-Electro-Mechanical System) switches have been receiving increasing attention. Such advantages render RF MEMS switches an attractive alternative to PIN or FET RF switches [1]. RF MEMS switches are widely used in the fields of aerospace [2], radar [3], mobile communications [4], tunable filters [5], phase shift networks [6], and other notable fields. These switches must satisfy stringent reliability requirements of failure-free operation over billions of cycles. However, due to the special structure thereof, such switches are frequently prone to failures.

Long term reliability still remains a matter of concern in the commercial utilization of RF MEMS switches [7]. The reliability of RF MEMS switches is affected by mechanical and electrical phenomena such as stiction [8], fatigue [9], creep [10], impact force [11], residual stresses [12], and dielectric charging [13]. Among all these reliability issues, the presence of impact force is a major problem. Due to the mechanical nature thereof, the impact force during the suction phase of the switch is considerably high, causing the beam membrane to be prone to failure [14]. Although significant efforts have been made to develop materials that can maintain high switching speeds while keeping structural failures low, high impact force remains one of the leading causes of device failure [15]. For capacitive RF MEMS switches, the higher the impact force when the top and bottom electrodes of the switch come into contact, the faster the impact velocity. This results in more damage to the beam of the switch, which leads to a shorter switch life. In practice, there is always a trade-off between impact velocity and pull-in time. The variation of the fall time and impact velocity with the applied voltage was given in a previous study [11]. Common methods of reducing impact velocity include tailoring the shape of the actuation pulse [15], applying step voltage [16], and using charge drive [17]. A number of researchers have achieved significant results by adjusting the shape of the driving pulse to reduce the impact velocity [18]. Despite such efforts, the customized pulse technique can only be applied in relatively slow switches with switching times Ts ≥ 10 us, requiring sufficient time to form an effective pulse train [19]. The use of a charge drive from a constant current source to reduce the impact velocity in previous research [19] resulted in an 80% reduction in the impact velocity with a bias resistance of 33 MΩ, but caused a 182% increase in the switching time.

In the present study, a new method was established for reducing the impact velocity of RF MEMS capacitive switches while keeping the pull-in time essentially unchanged. A new shunt capacitive MEMS switch was designed based on the support pillars being added to both sides of the signal line of the conventional MEMS switch. The effect of the positioning of the support pillars on the pull-in voltage was analyzed, and the effects of different positions and heights of the support pillars on the electromechanical and electromagnetic performance of the MEMS switch were simulated and compared. The new structure proposed in this paper can be used in satellite, mobile phone communication, radar, and other applications requiring high life.

## 2. Proposed MEMS Switch Model

### 2.1. Working Principle

**Figure 1:**
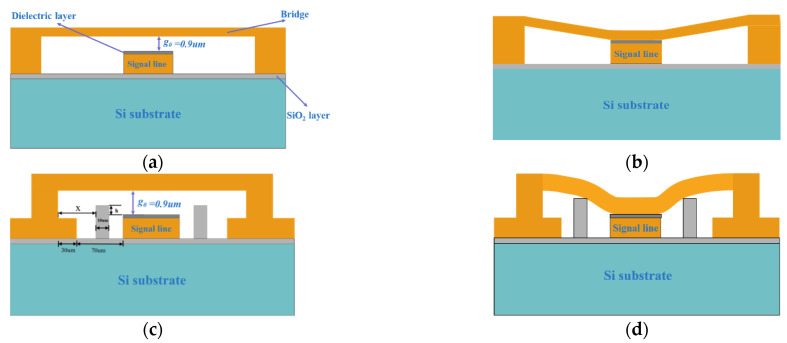
(**a**) Simplified capacitive RF MEMS switch OFF, (**b**) RF MEMS Switch ON, (**c**) The up state of the new RF MEMS switch, (**d**) The down state of the new RF MEMS switch.

The simplified capacitive RF MEMS switch structure is shown in Figure 1a. The shunt capacitive RF MEMS switch beam is suspended above the signal line at $g_{0}$ and connected to the left and right sides of the ground line through anchor points. When no driving voltage is applied to the switch, the capacitance between the bridge and the signal line is small, which can be described as denoted in Equation (1) [20]. The microwave signal can hardly be coupled to ground through such capacitance and thus propagates naturally in the waveguide, and the switch is on.

(1) $$C_{up}=\frac{\varepsilon_{0}A}{g_{0}+t_{d}/\varepsilon_{r}}$$

where g_0_ is the initial distance of the air gap between the top-electrode and the insulating medium; *A* is the area of driving electrode; $t_{d}$ is the thickness of the insulating medium film; $\varepsilon_{0}$ is the dielectric constant of air; and $\varepsilon_{r}$ is the relative dielectric constant of the insulating medium film material.

When a driving voltage is applied between the beam and the bottom-electrode, the bridge is pulled down by the electrostatic force. The capacitance between the bridge and the signal line increases substantially. When the applied driving voltage is greater than a certain critical limit voltage, the electrostatic force overcomes the mechanical stress limit of the beam, causing the bridge to collapse rapidly into contact with the dielectric layer. Here, the microwave signal is coupled to ground through such capacitance, which can be described as denoted in Equation (2) [20]. The signal cannot be transmitted here and the switch is off, at which point the critical voltage is referred to as the pull-in voltage ($V_{p}$). We define the time needed for the top electrode to reach the dielectric from its up state position as the pull-in time, and the velocity with which the top electrode lands on the dielectric as the impact velocity.

(2) $$C_{down}=\frac{\varepsilon_{0}\varepsilon_{r}A}{t_{d}}$$

### 2.2. Figures, Tables and Schemes

**Figure 2:**
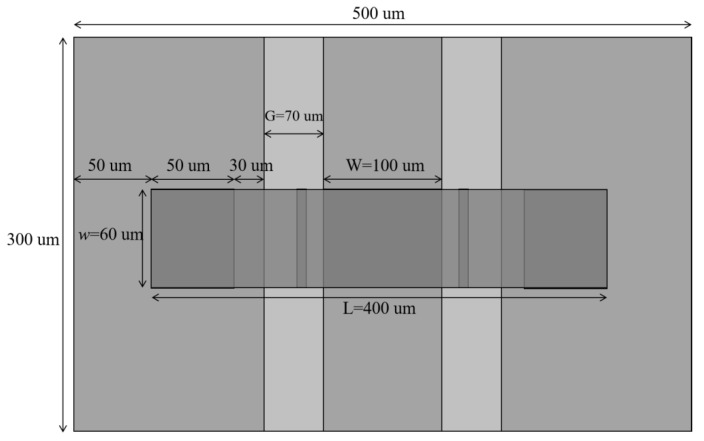
MEMS capacitive parallel switch top view.

Parallel MEMS switches are typically integrated in a coplanar waveguide (CPW) or microstrip lines, being a surface strip transmission line formed by a central signal line and two ground electrodes extending in parallel on the immediate sides thereof. The signal line and the ground electrode are located on the same surface of the substrate. The center of the signal line is covered with a dielectric layer with a thickness of $t_{d}$ and a dielectric constant of $\varepsilon_{r}$. To improve the adhesion of the metal to the substrate, a 1 um thick SiO_2_ layer was added between the substrate and the CPW transmission line. The length of the beam is L, the width is w, and the thickness is t. The width of the signal line is W and the gap is G. The top view of the capacitive parallel switch is shown in Figure 2.

**Figure 3:**
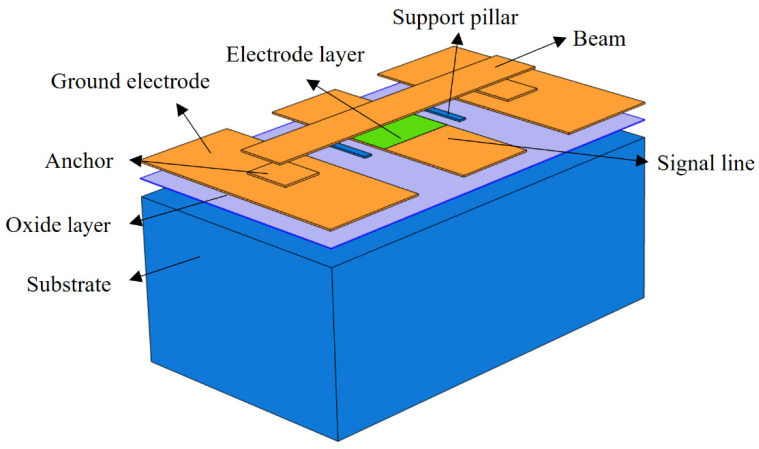
Schematic view of the proposed RF MEMS device.

The modified structure of the new MEMS switch is shown in Figure 1c,d and Figure 3. Two symmetrical support pillars, which are made of silicon, are added on both sides of the signal line. Although similar to the working principle of the conventional MEMS switch, the difference is that, during the process of applying driving voltage to pull-down the bridge, the bridge first contacts the support pillars to slow down the descent of the bridge before being pulled onto the dielectric layer. The equivalent elastic coefficient of the beam membrane becomes larger and the impact velocity decreases after the beam contacts the support pillars.

The new MEMS switch designed in the present study has high resistance silicon with a relative dielectric constant of 11.9 and a thickness of 300 um as the substrate. The CPW transmission lines, anchors, and the bridge are all made of gold. G/W/G = 70 um/100 um/70 um; the characteristic impedance is 50 Ω. The dielectric layer uses silicon nitride with a thickness of 0.1 um. The material of the support pillars is silicon with a width of 60 um, which should be no less than the width of the beam film in order to effectively support the beam film. There is also a 1 um thick silica buffer between the substrate and the CPW transmission line. The beam film length of the switch is l = 300 um, the thickness t is 2 um, and the gap $g_{0}\;$is 0.9 um. The material parameters of each component of the switch are shown in Table 1.

### 2.3. Proposed Pull-In Voltage Model

**Figure 4:**
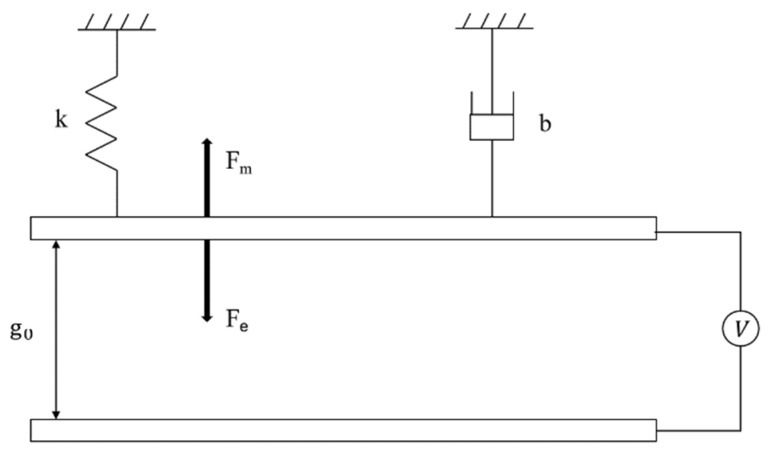
MEMS Switch Parallel Plate Equivalent Model.

For capacitive MEMS switches as shown in Figure 4, an electrostatic force is generated when the driving voltage is applied between the beam membrane and the pull-down electrode, which can be simplified to a parallel-plate capacitance equivalent model.

The pull-in voltage of MEMS switch $V_{p}$ can be expressed as follows [21]:

(3) $$V_{p}=\sqrt{\frac{8k_{m}g_{0}{}^{3}}{27\varepsilon_{0}A}}$$

where$\;\varepsilon_{0}$ is the dielectric constant in vacuum; and A is the area of the driving electrode. The formula for calculating the switching elastic coefficient $k_{m}\;$can be obtained as follows:

(4) $$k_{m}=32Ew\left(\frac{t}{l}\right)\frac{1}{8{\left(\frac{x}{l}\right)}^{3}-20{\left(\frac{x}{l}\right)}^{2}+14\left(\frac{x}{l}\right)-1}$$

where *E* is the Young’s modulus of the beam membrane material; *w* is the width of the beam; *t* is the thickness of the switch; and *l* is the length of the beam membrane.

When the load is uniformly applied to the central 1/3 length range of the solid-supported beam, the elasticity coefficient of the switch at this time can be obtained as denoted in Equation (5):

(5) $$k_{m}=32Ew{\left(\frac{t}{l}\right)}^{3}\times \frac{27}{49}$$

**Figure 5:**
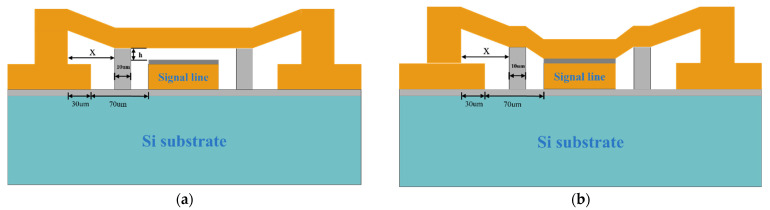
(**a**) MEMS Switch Membrane Equivalent Length Model, (**b**) MEMS Switch Equivalent Pull-in Model.

Two support pillars were added to the new MEMS switch. When the beam touches the support pillars during the pull-down process, the support pillars act as the anchor points of the beam, and the length between the support pillars can be equated to the length of the beam membrane. The gap between the beam membrane and the electrode is reduced from the original $g_{0}\;$to *h*. The equivalent beam membrane length l is reduced so that $k_{m}\;$ increases, rendering the beam membrane more difficult to pull-down. The distance between the support pillars and the anchor point is x, as shown in Figure 5.

Then, for the equivalent model, the length of the new beam film is:

(6) $$l^{\prime }=l-2\left(x+10\right)$$

Thus, Equations (3) and (5) can be updated as:

(7) $${k_{m}}^{\prime }=32Ew{\left(\frac{t}{l-2\left(x+10\right)}\right)}^{3}\times \frac{27}{49}$$

(8) $${V_{p}}^{\prime }=\sqrt{\frac{256}{49}\frac{Ewg_{0}{}^{3}}{\varepsilon_{0}A}{\left(\frac{t}{l-2\left(x+10\right)}\right)}^{3}}$$

As such, for the new MEMS switch, the beam membrane pull-down process can be divided into two parts: the first part refers to before the beam contacts the support pillars, and the pull-in voltage and spring coefficient are the same as those of the conventional MEMS switch. The second part refers to after the beam contacts the support pillars, and the pull-in voltage and spring coefficient are related to the support pillars positioning x and height h, as shown in Equations (9) and (10):

(9) $$V_{p}\left(g\right)=\{\begin{array}{cc} \sqrt{\frac{8k_{m}g_{0}{}^{3}}{27\varepsilon_{0}A}} & \;,g<g_{0}-h\\ \sqrt{\frac{256}{49}\frac{Ewg_{0}{}^{3}}{\varepsilon_{0}A}{\left(\frac{t}{l-2\left(x+10\right)}\right)}^{3}}, & \;g\geq g_{0}-h \end{array}$$

(10) $$k_{m}\left(g\right)=\{\begin{array}{c} 32Ew{\left(\frac{t}{l}\right)}^{3}\times \frac{27}{49},\;g<g_{0}-h\\ 32Ew{\left(\frac{t}{l-2\left(x+10\right)}\right)}^{3}\times \frac{27}{49},\;g\geq g_{0}-h \end{array}$$

where g is the descent displacement of the beam membrane; x is the positioning of the support pillars; $g_{0}$ is the distance between the beam membrane and the dielectric layer; and h is the height of the support pillars. g < g_0_ − *h* indicates that the beam membrane is not in contact with the support pillars, and $g\geq g_{0}-h$ indicates that the beam membrane is in contact with the support pillars.

**Figure 6:**
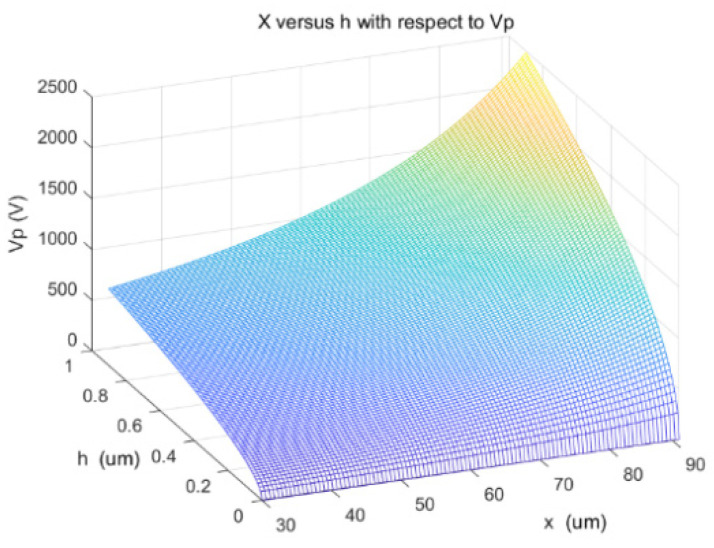
Relationship between position *x*, height *h*, and pull-in voltage.

Figure 6 depicts the relationship between the support post position x, the height h, and the pull-in voltage $V_{p}$. From Figure 6, the following observation can be made when the positioning of the support pillars is fixed: the larger the height h, the larger the gap between the beam film and the dielectric layer, and the larger the pull-in voltage $V_{p}$. When the position is increased, the equivalent beam film length decreases and $V_{p}$ gradually increases. Therefore, in order to ensure the pull-in voltage is within a reasonable range, the positioning and height of the support pillars need to be chosen reasonably.

## 3. Electromechanical Performance Analysis

Simulation software was used to model and simulate the new MEMS switch. Two support pillars were placed on both sides of the central signal line, as shown in Figure 3. The theoretical value of x ranged from 30 um to 90 um. The positions of the support pillars were on both sides of the signal line at 90 um and close to the ground line at 30 um. The height h of the support pillars was the equivalent gap $g_{0}$ after the beam membrane contacted the support pillars, and the theoretical value ranged from 0 um to 0.9 um. If the height h of the support pillars was 0 um, the pillars were not in contact with the beam membrane during the pull-down process. If the height h was 0.9 um, the pillars were always in contact with the beam membrane and the support pillars became the new anchor points.

**Figure 7:**
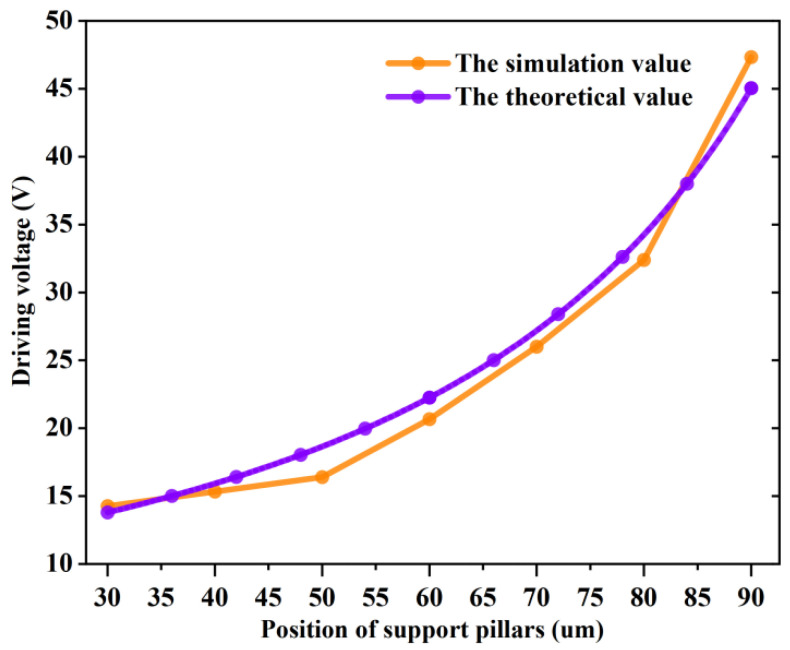
Influence of position of support pillars on pull-in voltage; the black curve is the theoretical value and the red curve is the simulation value.

Equation (8) shows that when the position x of the support pillars changed, the length of the equivalent beam membrane changed and the pull-in voltage $V_{p}$ would also change. Figure 7 depicts the relationship between the position x of the support pillar and the pull-in voltage $V_{p}$. The black curve is the theoretical value and the red curve is the simulated value. The distance between the top of the support pillars and the insulating dielectric layer was 0.75 um, but since the effect of the insulating dielectric layer is not considered in Equation (8), the initial distance needed to be added to the height of the insulating dielectric layer by 0.1 um. In the case of $g_{0}$ = 0.85 um, changing the value of x yielded a different pull-in voltage$\;V_{p}$.

As the position x increased, the pull-in voltage gradually increased, and the larger x was, the faster the pull-in voltage increased, reaching the maximum pull-in voltage at x = 90 um. Such findings could be attributed to a larger x, indicating that the support column is closer to the centerline. The stronger the hindering effect of the support pillars on the bridge, the larger the equivalent elasticity coefficient of the beam membrane. The more difficulties in pulling down the bridge, the higher the pull-in voltage of the bridge. The indication is that the positioning of the support pillars has a large influence on the driving voltage of the MEMS switch. For the MEMS switch, a suitable pull-in voltage can weaken the ion injection into the dielectric layer to cause shielding failure or adhesion failure [22]. Therefore, suitable support pillar positioning should be selected to keep the pull-in voltage within an acceptable range. The theoretical values in Figure 7 are basically consistent with the simulated data curves, indicating that the equivalent model is physically reasonable.

**Figure 8:**
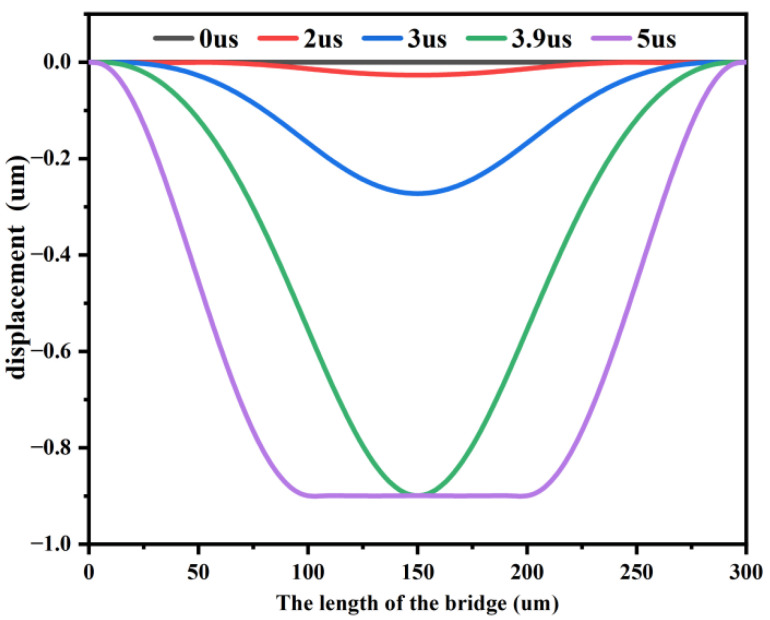
Shape of the beam at different instants of time during pull-down.

Figure 8 depicts the falling displacement of the beam membrane at different times. The displacement changed slowly until 3 us, and the falling displacement was about 1/3 of the gap g_0_ at 3 us. Subsequently, the displacement of the beam membrane increased rapidly and reached the maximum displacement at 3.9 us. The MEMS switch closed completely at 5 us. The simulation performance was as expected, and thus, the simulation software could be used for further analysis.

**Figure 9:**
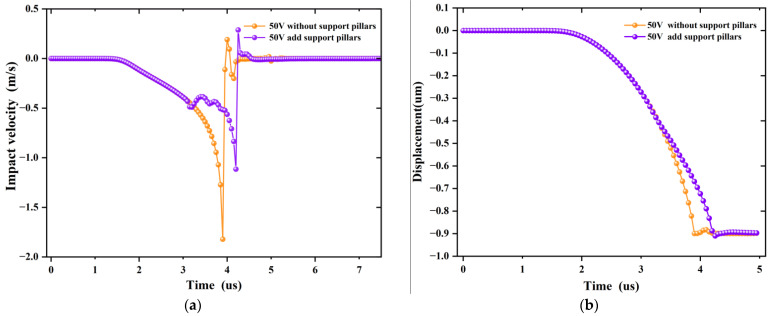
(**a**) Influence of adding support pillars on impact velocity, (**b**) Influence of adding support pillars on pull-in displacement.

Figure 9a depicts the impact velocity and descent displacement as a function of time with and without the addition of the support pillars, respectively, with a driving voltage of 50 V. The velocity and displacement of both were the same from 0 to 3 us, indicating that the beam membrane was not in contact with the support pillars. The velocity slowed down and fluctuated around 0.5 m/s after 1 us of contacting the support pillars, and then the velocity increased rapidly. The bridge contacted the dielectric layer at 4.2 us when the instantaneous impact velocity reached a maximum of 1.1 m/s. Compared with the conventional MEMS switch, the pull-in time was only slightly increased, but the impact velocity was significantly reduced. Figure 9b shows that there was a significant difference in the descent displacement after the beam membrane came into contact with the support pillars, which acted as a buffer for the descent of the bridge.

**Figure 10:**
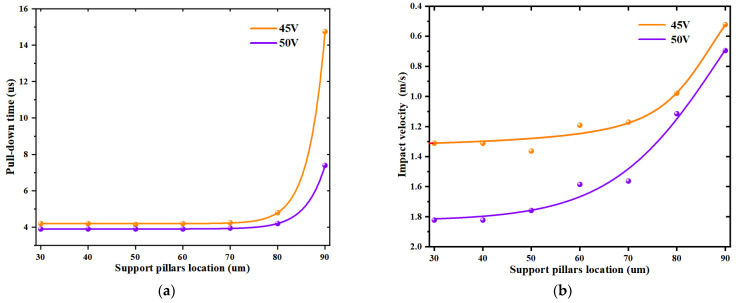
(**a**) Pull-in time at different positions of the support pillar, (**b**) Impact velocity at different positions of the support pillar.

Figure 7 indicates that when the positioning of the support pillars changed, the MEMS switch pull-in voltage also changed. The impact velocity and pull-in time were not only related to the positioning of the support pillars but also to the applied voltage. If the applied voltage was less than the pull-in voltage causing the switch to fail to close, the impact velocity and pull-in time did not exist. As such, a voltage three to four times higher than the pull-in voltage was applied to weaken the effect of different pull-in voltages in order to observe the effect of the change of the support pillar positioning on the impact velocity and the pull-in time. The simulations were performed by applying 45 V and 50 V, respectively, and the height of the support pillars was 0.75 um. Figure 10a shows the variation of the pull-in time by different positions of the support pillars, and Figure 10b shows the variation of the impact velocity by different positions of the support pillars.

The impact velocity and pull-in time of the conventional MEMS switch and the new MEMS switch were considerably close to each other because the obstruction of the beam membrane by the support pillars was considerably small until 30 um. Figure 10a shows that the pull-in time changed only slightly until 80 um, and the time increased significantly afterwards. Figure 10b shows that the impact velocity decreased slightly with the increase in the support pillar positioning until 60 um, while the impact velocity decreased significantly between 60 um and 80 um, compared with the conventional MEMS switch. From the aforementioned analysis, a conclusion can be drawn that the increase in the support pillar position led to an increase in the pull-in time and a decrease in the impact velocity. Therefore, a suitable position can be found where the pull-in time remains the same while the impact velocity decreases significantly. As an example, at *x* = 80 um, the closing time was 4.2 us and the impact velocity was 1.1 m/s. Compared with the pull-in time of 3.9 us and the impact velocity of 1.8 m/s without the support pillars, the closing time increased by 0.05% and the impact velocity decreased by almost 40%.

**Figure 11:**
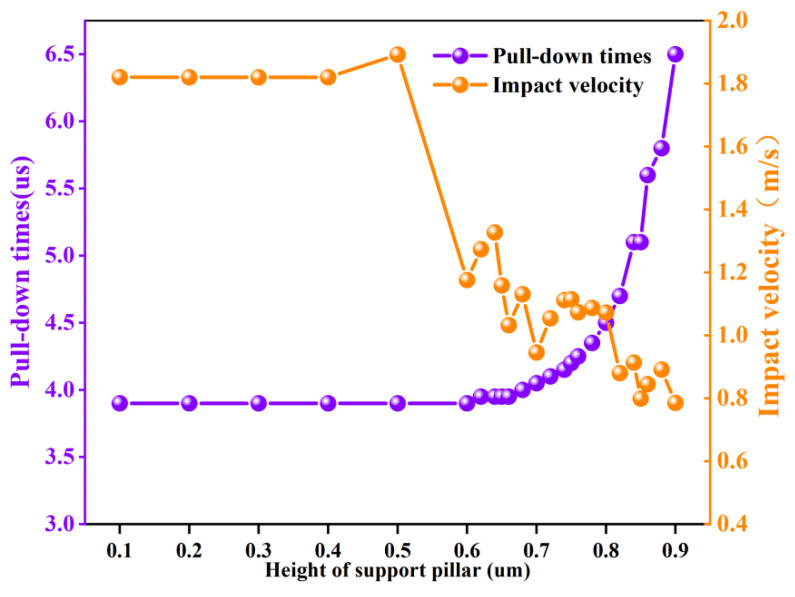
Effect of changing the height of the support pillars on impact velocity and pull-in time.

As shown in Figure 11, the simulation was repeated several times when the driving voltage was 50 V and the positioning of the support pillars was changed to 80 um to analyze the change of the positioning of the support pillars on impact velocity and pull-in time.

Since the beam membrane did not come into contact with the support pillars during the pull-down process when the height was less than 0.4 um, the impact velocity and pull-in time were basically the same as when no support pillars were added. After 0.5 um, the pull-in time increased gradually with the increase in height. Further, as the height of the support pillars increased, the increase in the pull-in time became increasingly larger. Because the instantaneous velocity change of the bridge contacting with the dielectric layer was significantly large, the simulation results of the impact velocity had some errors due to the limitation of the mesh division accuracy of the simulation. However, an observation can be made from Figure 11 that the overall change of impact velocity after 0.5 um exhibited a decreasing trend. A conclusion can be drawn that the higher the height of the support pillars, the longer the pull-in time and the smaller the impact velocity. Therefore, a suitable height can be found to make the best balance between pull-in time and impact velocity. For instance, for *x* = 0.8 um, the impact velocity was 0.8 m/s, which was 45% less than 1.8 m/s without the support pillars, and the pull-in time was 4.7 us, which was 0.2% more than 3.9 us without the support pillars.

From the aforementioned analysis, a conclusion can be drawn that the positioning and height of the support pillars had a significant influence on the impact velocity and the pull-in time of the MEMS switch. The closer the positioning of the support pillars to the signal line and the higher the height, the lower the impact velocity and the longer the pull-in time, which is not suitable for high frequency applications. Thus, choosing the right position and height is critical. The positioning and height of the support pillars were not only related to the impact velocity and pull-in time, but also to the electromagnetic performance of the MEMS switch.

## 4. Electromagnetic Performance Analysis

In the present study, an electromagnetic simulation model of a parallel-connected capacitive RF MEMS switch was constructed. Further, the positioning and height of the support pillars were scanned and optimized by means of simulation software to derive the effect thereof on the electromagnetic performance. A total of nine combinations of *x* = 70 um, 80 um, and 90 um, and *h* = 0.7 um, 0.8 um, and 0.9 um were simulated.

**Figure 12:**
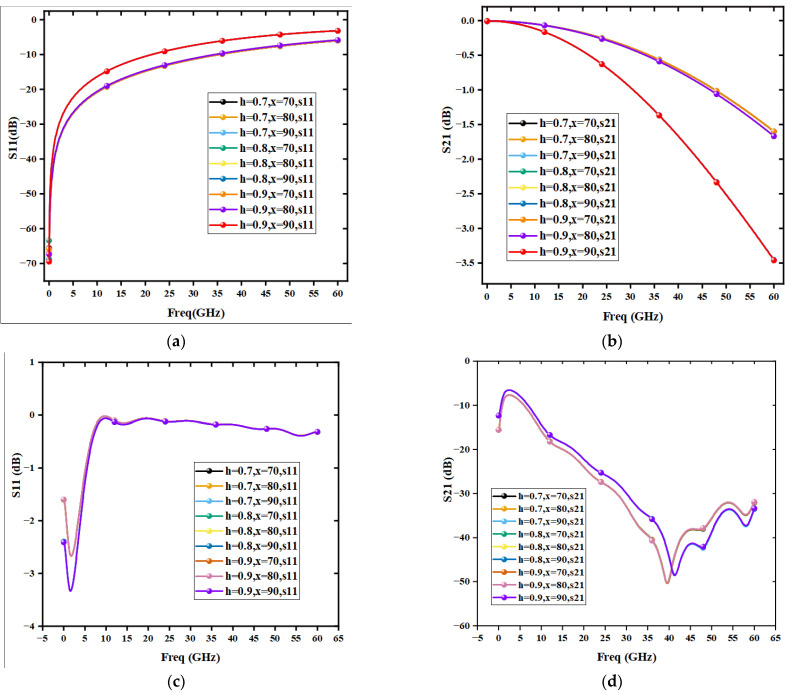
Variation of the position and height of the support pillars on the electromagnetic performance of the MEMS switch, (**a**) MEMS open-state S11, (**b**) MEMS open-state S21, (**c**) MEMS off-State S11, (**d**) MEMS off-state S21.

As shown in Figure 12, the curves of different heights at the same position approximately overlap, indicating that the height of the support pillars had basically no effect on the electromagnetic performance of the switch. Figure 12a,b shows that the curves of S11 and S21 at x  = 70 um and x = 80 um in the open-state approximately overlap, and the electromagnetic performance became worse at x = 90 um. Such results show that the increase in the position x of the support pillars decreased the electromagnetic performance of the MEMS switch. An observation can be made that the closer the support pillars are to the center signal line, the larger the S21 in the open-state and the smaller the S21 in the off-state. Such findings can be attributed to the contact between the support pillars and the signal line changing the equivalent capacitance of the MEMS switch, thereby degrading the electromagnetic performance of the switch. Thus, the actual support pillars should not be positioned too close to the center signal line. The influence of the height and position of the support pillars on the electromagnetic performance was considered to be optimal for the MEMS switch at x = 80 um and h = 0.8 um. The insertion loss was less than 0.5 dB up to 32 GHz, and the isolation was more than 50 dB at 40 GHz. Therefore, the effect of adding the support pillars on the electromagnetic performance of the MEMS switch was considerably small, and the suitable positioning of the support pillars could be selected to weaken the effect thereof. Overall, the proposed scheme of adding the support pillars is practical and feasible.

## 5. Conclusions

The present study provides a new method for reducing the impact velocity of the MEMS switch. Specifically, two symmetric support pillars were added under the bridge to buffer the beam during the falling process, thus reducing the impact velocity of the MEMS switch. The effect of adding the support pillars on the impact velocity and pull-in time of the MEMS switch was also analyzed, and the positioning and height of the support pillars were scanned and optimized. A conclusion can be drawn that the closer the support pillars are to the signal line and the higher the height, the lower the impact velocity and the longer the pull-in time. The electromagnetic performance of the switch was largely unaffected by the height of the support pillars. However, when the support pillars were considerably close to the signal line, the electromagnetic performance became considerably poor. The influence of the height and positioning of the support pillars on the impact velocity, pull-in time, and electromagnetic performance was found to be reduced by 40% from 1.8 m/s to 1.1 m/s at x = 80 um, h = 0.8 um, and 50 V applied voltage. The closing time increased from 3.9 us to 4.2 us, which was only 0.05%, the insertion loss was less than 0.5 dB up to 32 GHz, and the isolation degree was more than 50 dB at 40 GHz. The experimental data suggest that the RF MEMS switch designed in the present study has lower impact speed, shorter closing time, and better electromagnetic performance. Among other methods to reduce the impact velocity, the charge drive of the constant current source is used in the literature [2] to reduce the impact velocity by 80%. The switching time increased by 182%. In the literature [3], the impact velocity was reduced by 21.6% by modulating the actuation force with open-loop operations. We provided the method of adding support pillars, which reduced the impact velocity by 40% but increased the pull-in time by 0.05%.

However, the proposed new method is not perfect. The proposed switch reduces the impact velocity and increases the closing time at the same time, although the increased closing time is acceptable. In addition, manufacturing the proposed MEMS switch requires more than one process to manufacture the support column, which means that manufacturing this switch is technically complicated.

## Figures and Tables

**Table 1:** Dimensions of the proposed switch.

Component	Width (μm)	Depth (μm)	Height (μm)	Material	Dielectric Constant
Substrate	300	500	300	High Resistivity Silicon	11.9
Oxide layer	300	500	1	Silicon Dioxide	4.2
Anchor	60	50	1	Gold	-
Ground electrode	300	130	2	Gold	-
Signal line	300	100	2	Gold	-
Electrode layer	60	100	0.1	Silicon Nitrate	9.7
Beam	60	400	2	Gold	-
Support pillar	60	10	2.80	Silicon	4.5

## Data Availability

Data available on request from the authors.
